# Testing the Emergence of New Caledonia: Fig Wasp Mutualism as a Case Study and a Review of Evidence

**DOI:** 10.1371/journal.pone.0030941

**Published:** 2012-02-22

**Authors:** Astrid Cruaud, Roula Jabbour-Zahab, Gwenaëlle Genson, Stefan Ungricht, Jean-Yves Rasplus

**Affiliations:** 1 INRA-UMR Centre de Biologie et de Gestion des Populations, CBGP, (INRA/IRD/CIRAD/Montpellier SupAgro), Campus international de Baillarguet, Montferrier-sur Lez, France; 2 ETH Zurich, Zurich, Switzerland; American Museum of Natural History, United States of America

## Abstract

While geologists suggest that New Caledonian main island (Grande Terre) was submerged until *ca* 37 Ma, biologists are struck by the presence of supposedly Gondwanan groups on the island. Among these groups are the *Oreosycea* fig trees (*Ficus*, Moraceae) and their *Dolichoris* pollinators (Hymenoptera, Agaonidae). These partners are distributed in the Paleotropics and Australasia, suggesting that their presence on New Caledonia could result from Gondwanan vicariance. To test this hypothesis, we obtained mitochondrial and nuclear markers (5.3 kb) from 28 species of *Dolichoris*, used all available sequences for *Oreosycea*, and conducted phylogenetic and dating analyses with several calibration strategies. All our analyses ruled out a vicariance scenario suggesting instead that New Caledonian colonization by *Dolichoris* and *Oreosycea* involved dispersal across islands from Sundaland *ca* 45.9-32.0 Ma. Our results show that successful long-distance dispersal of obligate mutualists may happen further suggesting that presence of intimate mutualisms on isolated islands should not be used as a priori evidence for vicariance. Comparing our results to a review of all the published age estimates for New Caledonian plant and animal taxa, we showed that support for a vicariant origin of the island biota is still lacking. Finally, as demonstrating a causal relationship between geology and biology requires independent evidence, we argue that a priori assumptions about vicariance or dispersal should not be used to constrain chronograms. This circular reasoning could lead to under or overestimation of age estimates.

## Introduction

The New Caledonia archipelago (Grande Terre, Loyalty Islands, Isle of Pines, and a number of smaller islands) is isolated in the southwest Pacific. These islands are roughly located 1220 km east of Australia, 1700 km north of New Zealand, 1000 km south of the Solomon Islands, and about 400 km southwest of the Vanuatu archipelago [1]. New Caledonia, and especially Grande Terre, is known as a biodiversity hotspot due to its diverse, endemic, and threatened biota [2], [3]. Until recently, the high level of diversity, the old age of New Caledonia's geological basement and the presence of some ancient groups were taken as evidence that Grande Terre (hereafter shortly named “New Caledonia”) was a Gondwanan refuge where plants (e.g. *Amborella* [4], *Nothofagus* [5], *Araucaria* [6], *Ficus* [7]) and vertebrates (e.g. the Kagu flightless bird from the monotypic family Rhynochetidae [8]) have survived for more than 90 Myr [1]. However, recent geological evidence suggest that the New Caledonian region experienced complete submersion until *ca* 37 Ma [9], [10], [11] and no mainland area was above sea level between the Cretaceous and the Late Eocene [12]. This implies that the present day biota of New Caledonia must comprise neo-endemics evolved from Cenozoic transoceanic dispersers [1]. However, to explain an apparent mosaic assemblage of taxa of different ages, some biogeographers questioned a complete submersion of the island [13], [14] and some proposed that old taxa could have survived on ephemeral habitats until New Caledonia's emersion [15], [16]. Therefore, New Caledonia is immersed in a controversy over the age and origin of its biota.

Among the emblematic and putative Gondawanan groups occurring in New Caledonia are the fig trees (*Ficus*, Moraceae) and their associated pollinators (Chalcidoidea, Agaonidae) [7]. New Caledonia hosts 24 endemic *Ficus* species all belonging to the section *Oreosycea* (subgenus *Pharmacosycea*) and pollinated by wasps from the genus *Dolichoris* [17], some of them endangered, threatened by habitat destruction [18]. Corner [19] hypothesized that the Coral Sea area including today's main island of New Caledonia was the radiation centre of this group of monoecious fig trees. In New Caledonia, evolutionary radiation of *Oreosycea* figs as well as their pollinators were accompanied by high ecological and morphological disparity among species (habit, fruit position, leaf shape; head shape, mandible size) [19], [20]. *Oreosycea* fig trees and their pollinators are distributed in the Paleotropics and Australasia, suggesting vicariance resulting from the break-up of east-Gondwanaland [7] (see materials and methods and Table S1, for the detailed taxonomy and distribution). Moreover, available age estimates (*ca* 70-45 Ma for the figs [21] and 95-46 Ma for the wasps [22]) are contemporaneous with the opening of the Tasman Sea that started 83 Ma and ended 52 Ma [11], [23], [24]. Here, we propose to test whether a vicariance scenario could explain the presence of both partners on New Caledonia. Answering this question is of particular importance to debates concerning New Caledonia's history. First, figs are a major resources for tropical ecosystems [25], and one can suppose that the biogeographical history of numerous frugivores is linked to those of fig-pollinator mutualists. Second, pollinating fig wasps are mostly associated to a single species of *Ficus* and neither partner can reproduce without the other. Successful dispersal of obligate mutualisms requires to quickly find one another after independent dispersal [26]. Theoretically [27] and intuitively, mutualisms appear less prone to successful dispersion over long distance than single species. Consequently, they might be viewed as evidence of ancient vicariance. Moreover, testing a vicariance scenario may also shed light on the dispersal ability of mutualisms.

We obtained nuclear and mitochondrial DNA sequences (5.3 kb) from all major lineages of the genus *Dolichoris* and gathered all the *Oreosycea* sequences published so far. We conducted phylogenetic analyses on both datasets, though due to undersampling of fig species we only used the fig wasp dataset to conduct relaxed molecular dating analyses with several calibration strategies. Since the review by Grandcolas et al. [1], including 11 taxa, a great number of studies including New Caledonian groups have been published (see Table S4). We then conducted an up to date review of all dated, molecular-based phylogenies incorporating New Caledonian taxa and compared these age estimates to our results.

## Materials and Methods

### Taxonomic sampling and laboratory protocols

*Oreosycea* figs and their associated pollinators are distributed in the Paleotropics, Australasia (mostly New Caledonia and Papua) and South-East Asia [17], [19]. A few species are known from Africa and Madagascar [28] and only one species occurs in Australia [29]. The detailed taxonomy and distribution of *Oreosycea* fig trees is provided in Table S1. The section is divided into two subsections: *Glandulosae* and *Pedunculatae* [30]. The sub-section *Glandulosae* contains two groups of fig trees: the «*Ficus austrocaledonia* group» that comprises 27 species restricted to Pacific Islands (among which 24 are endemic to New Caledonia, 2 to Solomon Islands and 1 to Vanuatus), and the «*Ficus nervosa* group» that contains 23 species from India to the Solomon Islands. The Subsection *Pedunculatae* includes nine species, three of which are widely distributed from India and Continental Asia to Wallace line and Australia.

We included 28 species of *Dolichoris* representing three times the number of described species and about 33% of the world estimated diversity (Rasplus, unpublished). Twenty-one of these species are new to science (numbered sp. 01 to sp. 21). They pollinate 23 of the 59 *Oreosycea* known species. Sixteen *Dolichoris* species pollinate *Ficus* belonging to the *F. austrocaledonica* group, fourteen of which are endemic to New Caledonia. Nine *Dolichoris* species pollinate figs of the *F. nervosa* group and three species figs of the sub-section *Pedunculatae.* None of these species were endangered or protected species. Field studies have been funded by the French National Research Agency (ANR project “BioNEOCAL” to J.Y. Rasplus). Eight species belonging to the genera *Ceratosolen*, *Pegoscapus*, *Pleistodontes* and *Tetrapus* (Agaonidae) were used as outgroups [31]. To test the relationship of *Blastophaga* (s.s.) with the genus *Dolichoris* [31], *Blastophaga psenes*, the type species of the genus, was also included in our analyses. All material was collected alive and fixed in 95% EtOH. Vouchers are deposited at CBGP (Centre de Biologie pour la Gestion des Populations), Montferrier-sur-Lez, France. A list of all sampled species is given in Table S2. Extraction, amplification and sequencing protocols follow Cruaud et al. [31], [32]. Our final dataset was composed of six concatenated gene regions: 1) two nuclear genes: F2 copy of elongation factor-1a (EF-1a, 516 bp) and *wingless* (Wg, 403 bp); 2) two mitochondrial genes: cytochrome oxidase I (COI, 1449 bp) and cytochrome b (Cytb, 744 bp); and, 3) two nuclear ribosomal genes: 28S rRNA (D2–D3 and D4–D5 expansion regions, 1405 bp) and 18S rRNA (variable regions V3–5, 772 bp). All sequences were deposited in GenBank (Table S2). The *Oreosycea* phylogeny was reconstructed from all the sequences available in GenBank. The final dataset was comprised of ITS (891 bp), ETS (528 bp) and *G3pdh* (769 bp) sequences from 11 *Oreosycea* species and 8 outgroup species. *F. carica* which is pollinated by *Blastophaga psenes* was also included (Table S3).

### Phylogenetic and dating analyses

Protein-coding genes and hypervariable regions were aligned using ClustalW 1.81 default settings [33]. The alignment of rRNA sequences was based on secondary structure models [31], [34], [35]. The most appropriate model of gene evolution for each data subset (mitochondrial genes, EF-1a, rRNA stems and loops for wasps and ETS, ITS and G3pdh for figs) was identified using the Akaike information criterion implemented in MrAIC.pl 1.4.3 [36]. Phylogenetic trees were estimated using maximum likelihood (ML) and Bayesian methods. Analyses were conducted on a 150 cores Linux Cluster at CBGP. We performed ML analyses and associated bootstrapping using the MPI-parallelized RAxML 7.0.4 [37]. GTRCAT approximation of models was used for ML bootstrapping [38] (1000 replicates). Bayesian analyses of phylogenetic relationships and dating analyses were conducted using BEAST v 1.5.4 [39]. Indeed, recent studies have shown that models assuming independent molecular rates in adjacent branches perform better than those assuming a degree of rate autocorrelation especially on extended taxon sampling [40] (but see [41]). Due to insufficient sampling in the fig dataset, dating analyses were conducted only on the fig wasp dataset. Several calibration strategies have been examined to assess their effect on estimating the age of New Caledonian colonisation. Two Agaonid fossils belonging to the genera *Tetrapus* and *Pegoscapus* dated to the Burdigalian (20.4-16.0 Ma) have been described from the Dominican amber [42]. We first used these fossils to specify prior age distributions for the corresponding nodes (crown groups) by using 1) uniform (15.0–30.0 Ma), 2) normal (mean = 20; stdev = 3.0; 95% highest posterior density intervals (HPD) = 15.0–24.9 Ma) and 3) lognormal (offset = 15; log(mean) = 1.0; log(stdev) = 1.0; 95% HPD = 15.5–29.1 Ma) distributions successively. We then combined these fossil calibrations with geological information by specifying priors for the node grouping taxa endemic to Vanuatu islands. We implemented two distributions: 1) a lognormal prior distribution (offset = 0; log(mean) = 1.0; log(stdev) = 1.0; 95% HPD = 0.5–14.1 Ma) and 2) a normal prior distribution (mean = 2.0; stdev = 0.5; 95% HPD = 1.18–2.82 Ma). The shapes of the distributions used allowed for different degrees of uncertainty in fossil estimates, geological estimates and timing of island colonisation, which may impact the results. With the exception of a Yule tree prior, default priors were used for all parameters. Two runs of 60 000 000 generations were performed with sampling every 6000 generations. The two separate runs were then combined using LogCombiner ver. 1.5.4. We ensured convergence for each parameter using both TRACER ver. 1.5 and AWTY [43]. Following the removal of 10% burn-in, the sampled posterior trees were summarized using TreeAnnotator ver. 1.5.4 to generate a maximum clade credibility tree and calculate the mean ages, 95% highest posterior density intervals and posterior probabilities (PP).

### Review of studies including New Caledonian taxa

We reviewed the literature on dated, molecular-based phylogenies incorporating New Caledonian taxa in order to assess whether biological data contradict or agree with geological evidence of a complete submersion of New Caledonia until *ca* 37 Ma. As often as possible, and when available or applicable, we tried to report both mean stem and crown ages with their 95% highest posterior density (HPD) intervals. Indeed, one must keep in mind that the age of the stem group is the time of divergence of the given group from its sister taxon and the age of the crown group is the time of the deepest bifurcation within the given group [44]. Therefore, a colonisation event can be inferred at any time between the stem and the crown ages and the most conservative window of possible colonisation times is given by the upper 95% HPD interval of the first estimate and the lower 95% HPD interval of the second estimate (e.g. [45]). To be conservative, when authors reported variation on age estimates, we always kept the oldest estimate. All studies that used the emergence of New Caledonia (*ca* 37 Ma) as calibration point were discarded.

## Results

### Phylogenetic and dating analyses

**Figure 1:**
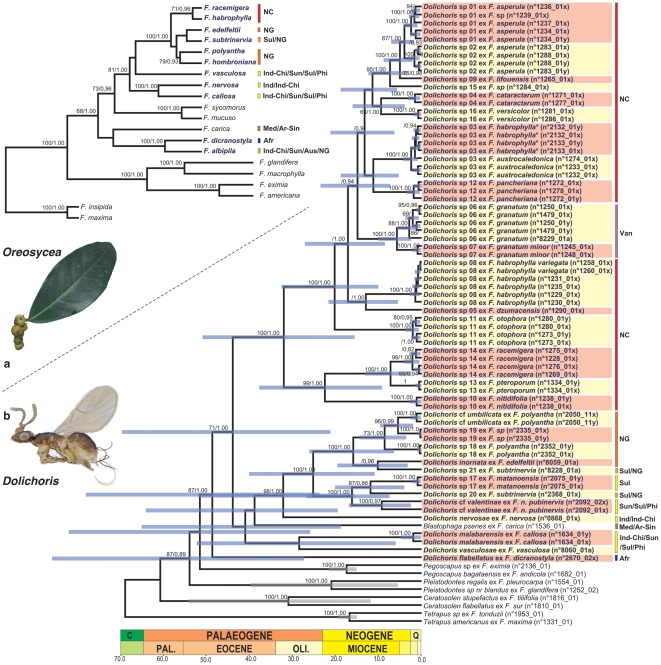
Maximum clade credibility trees obtained for *Oreosycea* fig trees (a) and chronogram showing the timing of evolution of their pollinating wasps (b). The chronogram showing the timing of evolution of *Dolichoris* fig wasps is derived from the sixth calibration set shown in Table 1. Grey bars around mean node ages (Ma) indicate the 95% HPD intervals. The geological time-scale is shown at the bottom. Bootstrap supports (higher than 65) and posterior probabilities (higher than 0.90) are indicated at nodes. The delineation of biogeographical regions follows Kreft [88] Afr: Africa, Ar-Sin: Arabo-Sindic; Aus: Australia, Ind-Chi: Indo-China, Ind: India, Med: Mediterranean; NC: New Caledonia, NG: New Guinea, Phi: Philippines, Sul: Sulawesi, Sun: Sundaland, Van: Vanuatu. Stars indicate probable misidentifications of host *Ficus* in Genbank. *Dolichoris* and *Oreosycea* species are highlighted in bold blue.

Bayesian and ML tree topologies were similar, only differing in the support of few clades and we arbitrarily chose to map node support values on the bayesian topologies (Figure 1).

All *Dolichoris* species clustered in a strongly supported clade (BP = 87; PP = 1.00). The Afrotropical species *D. flabellatus* was recovered sister to the remaining *Dolichoris* with strong support (BP = 71; PP = 1.00). The next node should be considered a polytomy of five subclades as supports for relationships are weak: 1) *Blastophaga psenes* (Arabo-sindic, Mediterranean); 2) *D. malabarensis*; 3) *D. vasculosae* (both Indo-China, Sundaland, Sulawesi, Philippines); 4) the pollinators of the *F. nervosa* group (Indo-China, Sundaland, Sulawesi, Philippines, New Guinea); and 5) *Dolichoris* from New Caledonia and Vanuatu. *Dolichoris* species from Vanuatu and Loyalty Islands are deeply nested within a strongly supported clade of *Dolichoris* endemic to Grande Terre (BP = 100; PP = 1.00).

*Oreosycea* was not recovered as monophyletic but instead formed two groups, a result already observed by Weiblen [46] and Rønsted et al. [47]. The *F. albipila* group (*F. albipila* (Indo-China, Sundaland, New Guinea, Australia) and *F. dicranostyla* (Africa)) appeared only distantly related to the remaining *Oreosycea* (BP = 81; PP = 1.00). *F. nervosa* (Indo-China), *F. callosa* (Indo-China, Sundaland, Sulawesi, Philippines) and *F.vasculosa* (Indo-China, Sundaland, Sulawesi, Philippines), were recovered sister to a well supported clade grouping species from New Caledonia, New Guinea and Sulawesi. BEAST results showed that all gene regions greatly deviate from a strict clock model (ucld.stdev greater than 1.0 for each marker) and that there is no strong evidence of rate autocorrelation in our phylogeny (covariance values spanning zero). As expected, fossil and geological calibrations chosen as well as the method for applying constraints to the nodes have a great impact on divergence time estimates (Table 1). The less informative the priors (uniform), the wider the credibility intervals of the posterior estimates. A normal distribution assuming a colonisation of Vanuatu Archipelago just after the emergence of the present islands *ca* 2 Ma [48] gives younger estimates (about 10 Myr younger). The split between New Caledonian *Dolichoris* and their closest relatives was estimated to a mean age ranging from 54.9 to 31.3 Ma. The most recent common ancestor of the New Caledonian diversification was estimated to a mean age ranging from 40.3 to 22.8 Ma. The colonisation of New Guinea occurred later with a mean stem age ranging from 32.2 to 18.4 Ma and a mean crown age ranging from 21.1 to 11.9 Ma. All these estimates were in agreement with previous results based on independent datasets [22].

**Table 1:** Results of dating analyses using different calibration strategies.

Calibration strategies	crown *Dolichori*s	stemNew Caledonia	crownNew Caledonia
**Fossils: crown ** ***Pegoscapu*** **s & ** ***Tetrapus***			
1) uniform priors	60.8(97.1-30.6)	54.9(87.1-28.4)	40.3(64.5-20.7)
2) normal priors	52.6(80.0-26.0)	47.4(74.0-23.9)	35.0(55.8-15.7)
3) lognormal priors	53.0(83.6-24.5)	47.4(76.5-23.6)	35.8(56.1-17.5)
**Fossils and islands** **(node grouping species endemic to Vanuatu)**			
4) Fossil uniform priors & islands lognormal priors	55.7(86.9-27.0)	50.1(79.4-25.5)	36.6(58.0-18.3)
5) Fossil normal priors & islands lognormal priors	50.8(78.7-25.5)	45.9(71.3-23.0)	33.5(53.6-15.7)
6) Fossil lognormal priors & islands lognormal priors	48.6(78.0-23.7)	43.9(69.6-21.2)	32.0(50.9-15.5)
7) Fossil uniform priors & islands normal priors	39.3(54.9-24.0)	35.0(48.1-21.4)	25.6(36.6-14.5)
8) Fossil normal priors & islands normal priors	34.7(51.67-19.9)	31.3(46.2-18.4)	22.8(35.4-12.0)
9) Fossil lognormal priors & islands normal priors	35.5(51.0-21.4)	32.0(45.7-19.1)	23.2(34.6-13.4)

	stemNew Guinea	crownNew Guinea	crown *Pegoscapus*
**Fossils: crown ** ***Pegoscapu*** **s & ** ***Tetrapus***			
1) uniform priors	32.2(51.6-15.2)	21.1(34.6-9.07)	20.6(28.2-15.0)
2) normal priors	27.4(43.0-12.8)	17.7(28.3-7.60)	19.1(25.0-13.8)
3) lognormal priors	27.0(43.3-11.0)	17.9(30.7-7.17)	17.6(21.9-15.1)
**Fossils and islands** **(node grouping species endemic to Vanuatu)**			
4) Fossil uniform priors & islands lognormal priors	29.0(46.0.6-13.2)	18.4(31.5-7.84)	19.9(27.5-15.0)
5) Fossil normal priors & islands lognormal priors	26.4(41.8-12.5)	16.5(27.3-6.53)	18.5(23.9-12.7)
6) Fossil lognormal priors & islands lognormal priors	25.2(40.1-10.9)	15.9(26.1-6.43)	17.4(21.5-15.1)
7) Fossil uniform priors & islands normal priors	19.8(27.8-12.0)	12.4(18.9-6.30)	18.3(24.5-15.0)
8) Fossil normal priors & islands normal priors	18.2(27.1-9.93)	11.7(19.0-5.87)	17.0(23.1-11.4)
9) Fossil lognormal priors & islands normal priors	18.4(27.7-10.9)	11.9(18.4-6.35)	16.9(20.0-15.1)

	crown*Tetrapus*	stemVanuatu	crownVanuatu
**Fossils: crown ** ***Pegoscapu*** **s & ** ***Tetrapus***			
1) uniform priors	18.2(24.5-15.0)	19.6(31.5-9.56)	8.44(15.1-3.02)
2) normal priors	16.8(22.9-10.7)	16.05(26.7-8.27)	7.06(12.7-2.52)
3) lognormal priors	16.9(19.9-15.1)	14.4(27.2-9.14)	7.25(12.6-2.87)
**Fossils and islands** **(node grouping species endemic to Vanuatu)**			
4) Fossil uniform priors & islands lognormal priors	17.6(22.9-15.0)	17.4(27.6-8.89)	6.50(11.2-2.33)
5) Fossil normal priors & islands lognormal priors	16.2(22.3-10.3)	15.6(24.9-7.45)	5.76(10.1-2.19)
6) Fossil lognormal priors & islands lognormal priors	16.8(19.5-15.1)	14.9(23.5-7.29)	5.74(10.1-2.45)
7) Fossil uniform priors & islands normal priors	16.8(20.5-15.0)	11.6(16.8-6.84)	2.62(3.39-1.82)
8) Fossil normal priors & islands normal priors	14.4(20.2-8.45)	10.5(15.9-5.94)	2.57(3.34-1.75)
9) Fossil lognormal priors & islands normal priors	16.5(18.7-15.1)	10.9(16.4-6.09)	2.62(3.38-1.83)

Mean age estimates (Ma) with 95% highest posterior density (HPD) intervals are given for selected nodes in the phylogeny. Details about the prior age distribution assumed in each case are provided in the method section.

### Review of studies including New Caledonian taxa

**Figure 2:**
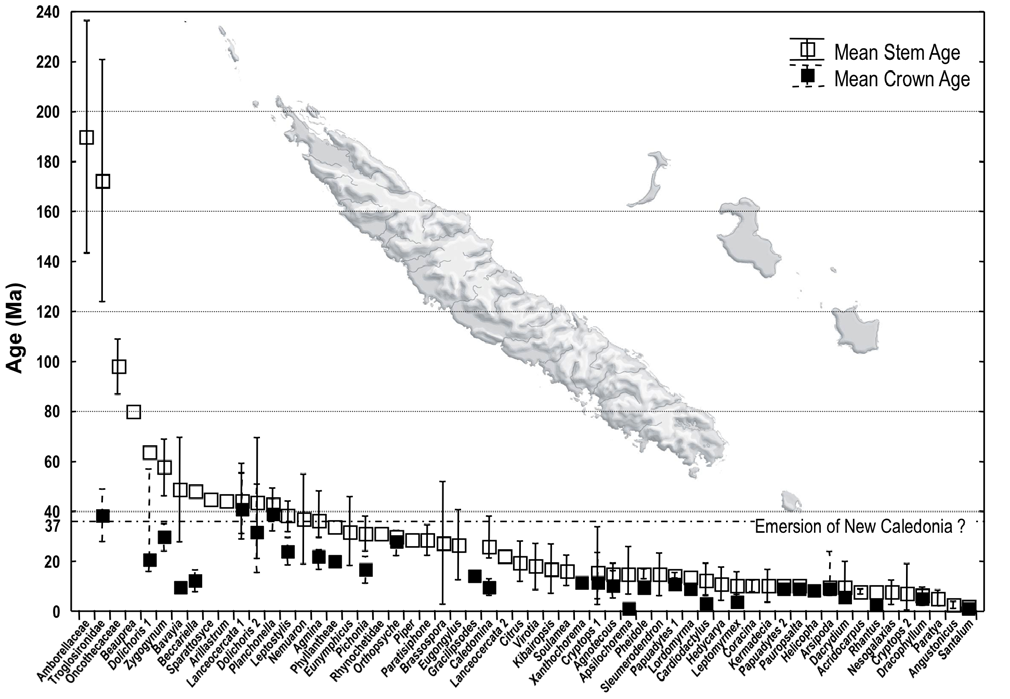
Review of the divergence time estimates for 50 New Caledonian clades. Taxon names refer to the list provided in Table S4. *“Dolichoris 2*” refers to the present study.

Our review includes 47 studies focusing on 54 different taxa (6 vertebrates, 24 arthropods, 24 plants) with different levels of endemicity (species, genus, family) (see Table S4 for details). Figure 2 summarizes the estimates of crown and/or stem divergence times obtained for each taxon. In about 75% of the groups in which divergence ages have been estimated using different markers, dating methods, and calibration points, both crown and stem mean ages postdate New Caledonia emergence (*ca* 37 Ma). About 16% of the groups had mean stem ages that predate New Caledonia emergence but their mean crown ages date back at most to 41.1 Ma. For five groups, the literature only reports stem ages, and three of them, namely *Amborella trichopoda*, *Oncotheca balansae*, and *Beauprea montana*, exceed 80 Ma.

## Discussion

### Timing of arrival in New Caledonia

Vicariance hypothesis predicts that the stem *Dolichoris* estimate should be older than the separation of Zealandia from Gondwana *ca* 80 Ma [23]. However, our dating analyses revealed that the split between New Caledonian species and their closest relatives is more recent. With the exception of the most uninformative calibration strategy (single calibration with uniform distribution, [49], [50]), even the upper confidence limit of our age estimate (76.5 Ma) rules out the latter hypothesis. Therefore, *Dolichoris* and their host figs have probably colonised New Caledonia by dispersal and not by lineage survival on islands formed by tectonic breakup of Gondwana.

A vicariance scenario is not supported by any of the age estimates published to date. Indeed, a post Bartonian (*ca* 40.3 Ma) colonisation cannot be rejected for any extant taxa (Figure 2 and Table S4), corroborating the observations of Grandcolas *et al.* (2008). Moreover, in most groups, both the colonisation and the diversification are relatively recent, contradicting the view that the native biota of New Caledonia is primarily a product of a long isolation [51], [52].

Even if three hypothetical vicariant groups, *Amborella trichopoda*, the subfamily Oncothecaceae and the genus *Beauprea*, seem to challenge other evidence, the available data appear uninformative with regard to their New Caledonian history. Although the genus *Beauprea* and the subfamily Oncothecaceae contain 13 and two species respectively, the published estimates are only based on one extant species per taxon allowing the estimation of stem ages only. Therefore, including other extant species, fossils or yet undiscovered species belonging to these groups is required to draw more compelling conclusions. The monotypic family Amborellaceae is likely a product of one of the oldest lineage-splitting events in all angiosperms [4] and has an extremely restricted present-day distribution. It seems therefore unlikely that it is the sole member of its lineage ever to have evolved [53]. Consequently, this phylogenetic relict, often referred to when arguing for a Gondwanan origin of the New Caledonian biota, should not be considered as informative [1], [54].

Some authors argued that the age estimates for the New Caledonian colonisation are underestimated because analyses are based on inaccurate fossil or biogeographic calibration priors [16], [55]. For example, Heads [16], [55] underlines that authors should not rely on the fossil record to set maximum age constraint on a given clade. It has been shown that using fossil evidence as «hard» minimum bounds (point calibrations) can result in underestimation of divergence times [49], [56]. However, the recently developed methods we used are able to incorporate calibration uncertainty using «soft» priors with parametric distributions [49], [57]. It has been also shown that taxa can be older than the land they inhabit (e.g. [58]), which is why using the formation of islands as hard maximum bounds is inappropriate in some cases. To date, *Dolichoris* species are known only from the southern part of the Vanuatu archipelago that emerged *ca* 2 Ma (Tanna and Anatom islands, [48]). For this reason, the use of a normal prior distribution centered at 2 Ma and covering a narrow range with 95% probability (1.18–2.82 Ma) for the node grouping Vanuatu endemics would appear justified. However, Vanuatu's ecosystems have formed during the last *ca* 25 Myr [59], [60] and we cannot rule out an older colonisation of the archipelago by the fig wasp mutualism. The biodiversity of the archipelago is still poorly known and endemics might have existed further north, on older islands. Therefore the use of a lognormal prior with a rigid minimum bound of zero and covering a wider range (0.5–14.1 Ma with 95% HPD), provides a more conservative calibration approach. This strategy resulted in 10 Myr older estimates for the New Caledonian colonisation (Table 1). This shows that a priori assumptions about the biogeographical history of lineages can have important effects on divergence time estimates. Even if geological events, such as the formation of islands, can offer plausible instances of maximum age bounds, these bounds must be chosen carefully. Using a too recent dispersal event as constraint on the origin of a given clade can result in underestimation of the age of all the lineages. Conversely and although this is advocated by some authors as the only accurate method to date New Caledonian colonisation [55], [61], constraining chronograms according to the ages of putative vicariance events would prevent vicariance falsification and could result in overestimation of divergence ages. Here, even under the most conservative dating strategy, our analyses show that the colonisation of New Caledonia by *Dolichoris* postdates the break-up of Gondwanaland. Moreover, even if some of the published analyses could be refined using more appropriate calibration strategies, it is striking that not one supports a vicariance scenario. Therefore, in the light of our study and the published evidence, we suggest that the most credible hypothesis is that New Caledonian biota is comprised of descendents of Cenozoic waif dispersers.

### Origin of New Caledonian colonisation

Most New Caledonian taxa share sister group relationships to taxa occurring in Australia, New Guinea, and New Zealand (see Table S4 and [55]). However, the origin of the lineages ancestral to New Caledonian endemics was rarely discussed in the studies we reviewed. Reconstruction of ancestral areas or inferences based on the closest outgroup node give ambiguous and contrasting results. This suggests that further analyses with representative sampling are still needed, to avoid reconstruction bias [54].

Interestingly, our results suggest an ancient colonisation of New Caledonia by *Oreosycea* and *Dolichoris* (*ca* 45.9-32.0 Ma) at a time when New Guinea was not yet formed and Sulawesi was divided in two islands [62]. The ancestors of the New Caledonian mutualists probably occurred somewhere in Sundaland and colonised independently New Caledonia and later Southern Sulawesi and New Guinea (*ca* 26.4-15.9 Ma). At least two colonisation route hypotheses can be proposed:

#### Hypothesis 1. From Sundaland directly to Australia and to New Caledonia

Although it cannot be definitively ruled out, this hypothesis is weakened by 1) the long distance between Sundaland and Northern Australia during the Eocene and early Oligocene, which makes transoceanic dispersal of figs and figwasps less likely (but see [54]); 2) the presence of only one *Oreosycea* species in Australia (*F. albipila*), furthermore not related to the New Caledonian clade [19], [47]. *F. albipila* is a widely distributed fig tree and its presence in Australia could be explained by its high dispersal capacities [29].

#### Hypothesis 2. From Sundaland to North Sulawesi, Philippines, Halmahera and New Caledonia through a series of shorter overwater dispersal events between islands

During the Eocene, distances between Sundaland, North Sulawesi, Philippines and Halmahera were relatively short [63] and may have enabled dispersal of the mutualists. About 45-43 Ma two subduction systems started and generated the Inner and the Outer (Vitiaz) Melanesian arcs [64]. All along the Vitiaz arc, an archipelago of volcanic islands probably occurred during the Eocene from north of present Papua to Vanuatu archipelago [65]. Further south, the Vitiaz arc was connected to New Caledonia by the Loyalty Ridge and the d'Entrecasteaux Ridge [11], [66]. Both the topology of our trees and the timing of New Caledonian colonisation suggest that these arcs have served as stepping stones for the eastward spread of *Dolichoris* and *Oreosycea.*

The stepping stones hypothesis predicts that close relatives of New Caledonian taxa should be present on the intermediate islands of the Vitiaz Arc. Indeed, two species of *Oreosycea* morphologically closely related to the New Caledonian species (*F. magwana* and *F. bubulia* [30], Table S1) occur in the Solomon Islands but we failed to collect and include them in our analyses. If the latter hypothesis is correct, their associated *Dolichoris* should be recovered sister to the New Caledonian clade by further phylogenetic analyses. Solomon Islands flora and fauna are still poorly known [51] and we suggest that taxa from this region, almost never included in studies, may be pivotal to our understating of the origin of New Caledonian fig mutualists. Once in New Caledonia, *Dolichoris* reached Vanuatu and Loyalties, ruling out the hypothesis that the current Loyalty islands have inherited their biota from previous ephemeral islands that dated back to Cretaceous [16].

### Co-dispersion of mutualists

*Ficus* are among the first trees to recolonize isolated islands after volcanic eruption [67], [68] and their fruits are dispersed by several hundreds of vertebrate species [25]. Among them pigeons [69], [70] and flying floxes [71], [72] are the most likely dispersers of figs in the Pacific region. *Ducula* and *Ptilinopus*, two genera of pigeons present in New Caledonia, retain seeds longer than do most other frugivorous birds [73]. *Pteropus* bats often fly long distances between islands and fig seeds have been demonstrated to pass intact through their guts [74].

Fig trees cannot reproduce without pollinating wasps and consequently have to be pollinated during their life spans to sucessfully colonized a new range. Life spans of fig trees are poorly known but they vary from a few tens of years to over 2000 years in banyan figs [75]. One of the oldest verified specimen of angiosperm is a fig tree (*F. religiosa*) planted in 288 BC in Sri Lanka [76]. Therefore agaonid pollinators have had to cross great distance within an extremely small window of time (at most a few hundred of years) in order to colonize New Caledonia and establish. Agaonidae are short-lived wasps [77], [78] blown by the wing to carry pollen between trees [79], [80]. They are capable of long distance dispersal and can reach fig trees separated by a few hundreds of kilometres of ocean or desert [81], [82]. In Borneo, *Dolichoris* and *Platyscapa* species are the agaonid wasps that flight the highest, reaching 60 meters, above the canopy [80]. As a result of turbulence and drag from tree crowns, wind-speeds increase with height above the canopy for the first few tens of metres [83]. Therefore, it is not surprising to find *Dolichoris* species as New Caledonian colonizers. The potential dispersal range of *Dolichoris* must be wide and distances of hundred of kilometers could be no barrier.

### Conclusion

In studies to distinguish between vicariance and/or dispersal in order to better explain the present-day distribution of biological groups, one must be cautious of circularity of argument [84]. This point seems of great importance here because the rejection of a complete submersion of New Caledonia is based on the presence of supposed Gondwanan groups on the island [14]. However, this circular logic is not based on scientific reasoning [1], [54]. Demonstrating causal relationships between geological phenomena and biological observations requires that geological and biological evidence are assessed independently [85]. Here, independently of any assumption about New Caledonian geological history, and using several calibration strategies, we provided evidence of an ancient dispersal for the fig wasp mutualism, a supposedly Gondwanan old group. This is the second example of a successful colonization of New Caledonia by mutualist partners. Indeed, dispersal to the island was already observed twice in the *Phyllanthus*/*Epicephala* obligate association [86], [87]. Therefore, while the presence of intimate mutualists on isolated islands might be viewed as evidence for vicariance our results highlight the fact that successful long-distance co-dispersal may occasionally happen. Finally, in reviewing the literature we showed that support for vicariant origins of any New Caledonian taxa is lacking. Therefore, biological data do not contradict but agree with geological evidence of a complete submersion of the island until *ca* 37 Ma.

## Supporting Information

Table S1 **Taxonomy and distribution of** ***Oreosycea*** **fig species.**
(DOC)

Table S2 **List of** ***Dolichoris*** **and outgroup species included in this study.**
(DOC)

Table S3 **List of** ***Oreosycea*** **and outgroup species included in this study**.
(DOC)

Table S4 **Details of studies included in the review of New Caledonia's biogeography.**
(DOC)
