# Supplementary material for: Testing the Emergence of New Caledonia: Fig Wasp Mutualism as a Case Study and a Review of Evidence
Source: PLoS One. 2012 Feb 22;7(2):e30941. doi: 10.1371/journal.pone.0030941 (PMC3285151; doi:10.1371/journal.pone.0030941)
Supplement: Table S1 — Taxonomy and distribution of Oreosycea fig species. (DOC) [file pone.0030941.s001.doc]

**Table S1: Taxonomy and distribution of *Oreosycea* fig species.**

*F. campicola* S. Moore, *F. comptoni* S. Moore, *F. cretacea* S. Moore, *F. edulis* Bureau, *F. hurlimannii* Guillaumin, *F. leptorachys* S. Moore, *F. oreadum* S. Moore, *F. longipes* Warb., *F. pallidinervis* Warb., *F. pseudomangiferifolia* Guillaumin, *F. punctulosa* Warb., *F. rigidifolia* Bureau, *F. semecarpifolia* Warb., *F. tanensis* G. Benn ex Seem*, F. trachyleia* Bureau are considered as synonym despite they have not been formely synonymised (S. Ungricht comm. pers.)

| **Subsection** | **Species group** | **Species** | **Distribution** | **Biogeography** |
| --- | --- | --- | --- | --- |
| Pedunculatae | F. albipila | *F. albipila* (Miquel) King (1888) | Thailand; Malaysia (Peninsula, Sabah); Indonesia (Java, Sumatra, Flores, Timor); Papua New Guinea; Australia | ori aus |
| Pedunculatae | F. vasculosa | *F. ampana* Berg (1986) | Madagascar | afr |
| Glandulosae | F. austrocaledonica | *F. asperula* Bureau (1872) | New Caledonia | aus |
| Pedunculatae | F. albipila | *F. assimilis* Baker (1890) | Madagascar | afr |
| Glandulosae | F. austrocaledonica | *F. auriculigera* Bureau (1872) | New Caledonia | aus |
| Glandulosae | F. austrocaledonica | *F. austrocaledonica* Bureau (1872) | New Caledonia | aus |
| Glandulosae | F. austrocaledonica | *F. barraui* Guillaum. (1954) | New Caledonia | aus |
| Pedunculatae | F. vasculosa | *F. bataanensis* Merrill (1906) | Philippines (Luzon, Negros, Palawan) | ori |
| Glandulosae | F. austrocaledonica | *F. bubulia* C.C. Berg (2002) | Solomon Islands (Big Nggela, Bougainville, Guadalcanal, Malaita, New Georgia, Small Nggela, Santa Isabel) | aus |
| Pedunculatae | F. vasculosa | *F. callosa* Willdenow (1798) | Andaman Islands; Burma; China; Indonesia (Java, Sumatra, Bali, Soemba, Timor, Moluccas, Sulawesi); Laos; Malaysia (Peninsula, Sarawak); Myanmar; Philippines; Vietnam | ori aus |
| Pedunculatae | F. albipila | *F. capillipes* Gagnepain (1927) | Cambodia; Vietnam; Laos; Thailand; Andaman Islands | ori |
| Glandulosae | F. nervosa | *F. carinata* C.C. Berg (2003) | Indonesia (Moluccas) | aus |
| Glandulosae | F. austrocaledonica | *F. cataractorum* Bureau (1872) | New Caledonia | aus |
| Glandulosae | F. austrocaledonica | *F. crescentioides* Bureau (1872) | New caledonia | aus |
| Pedunculatae | F. albipila | *F. dicranostyla* Mildbr. (1911) | Senegal; Guinea; Ivory Coast; Burkina Faso; Mali, Cameroon; Central African Republic; Ethiopia; Democratic Republic of Congo; Zambia; Uganda | afr |
| Glandulosae | F. austrocaledonica | *F. dzumacensis* Guillaumin (1926) | New Caledonia | aus |
| Glandulosae | F. nervosa | *F. edelfeltii* King (1887) | Indonesia (Irian Jaya); Papua New Guinea | aus |
| Glandulosae | F. nervosa | *F. gigantifolia* Merr. (1905) | Philippines (Bohol, Luzon, Mindanao, Samar) | ori |
| Glandulosae | F. austrocaledonica | *F. granatum granatum* G. Forst. (1786) | Vanuatu (Tanna, Aneityum, Ambrym, Eromanga) | aus |
| Glandulosae | F. austrocaledonica | *F. granatum* *minor* Corner (1960) | Vanuatu (Aneityum, Eromanga) | aus |
| Glandulosae | F. nervosa | *F. gratiosa* Corner (1960) | Indonesia (Sulawesi) | ori |
| Glandulosae | F. austrocaledonica | *F. habrophylla* G. Been ex Seem. (1860) | New Caledonia | aus |
| Glandulosae | F. nervosa | *F. hadroneura* Diels (1935) | Indonesia (Irian Jaya); Papua New Guinea (PNG, New Britain) | aus |
| Glandulosae | F. austrocaledonica | *F. heteroselis* Bureau (1872) | New Caledonia | aus |
| Glandulosae | F. nervosa | *F. hombroniana* Corner (1960) | Indonesia (Sulawesi, Moluccas, Irian Jaya); Papua New Guinea; Solomon Islands (Kolombangara, Bougainville, Malaita, Malaupaina, San Cristobal, Santa Cruz) | ori aus |
| Glandulosae | F. nervosa | *F. ihuensis* Summerh. (1919) | Papua New Guinea | aus |
| Glandulosae | F. nervosa | *F. illiberalis* Corner (1967) | Solomon Islands (San Cristobal, Guadalcanal, Kolombangara) | aus |
| Glandulosae | F. nervosa | *F. kjellbergii* Corner (1960) | Indonesia (Sulawesi) | ori |
| Glandulosae | F. austrocaledonica | *F. leiocarpa* ( Bureau) (1905) | New Caledonia | aus |
| Glandulosae | F. austrocaledonica | *F. lifouensis* Corner (1970) | New Caledonia (Lifou) | aus |
| Glandulosae | F. nervosa | *F. magnoliifolia* Bl. (1825) | Andaman Islands; Nicobar; Malaysia (Peninsula, Sarawak); Indonesia (Sumatra, Java, Sumbawa, Flores, Sulawesi); Philippines (Luzon, Leyte, Mindanao) | ori |
| Glandulosae | F. austrocaledonica | *F. magwana magwana* C.C. Berg (2002) | Solomon Islands (Big Nggela, Guadalcanal, Kolombangara, Malaita, New Georgia, Ranongga, Santa Isabel, Vella Lavella) | aus |
| Glandulosae | F. austrocaledonica | *F. magwana maragona* C.C. Berg (2002) | Solomon Islands (Santa Cruz) | aus |
| Glandulosae | F. austrocaledonica | *F. maialis* Bureau (1949) | New Caledonia | aus |
| Glandulosae | F. nervosa | *F. matanoensis* C.C. Berg (2003) | Indonesia (Sulawesi) | ori |
| Glandulosae | F. austrocaledonica | *F. microtophora* Corner (1975) | New Caledonia | aus |
| Glandulosae | F. austrocaledonica | *F. mutabilis* Bureau (1872) | New Caledonia | aus |
| Glandulosae | F. nervosa | *F. nervosa minor* (King) C.C. Berg | India; Sri Lanka | ori |
| Glandulosae | F. nervosa | *F. nervosa nervosa* Heyne ex Roth (1821) | India; Myanmar; China; Laos; Taiwan; Vietnam | ori |
| Glandulosae | F. nervosa | *F. nervosa pubinervis* (Blume) C.C. Berg (2003) | Indonesia (Java, Sumbawa, Flores, Timor, Sulawesi, Sumatra, Moluccas); Borneo ; Philippines; Taiwan | ori |
| Glandulosae | F. austrocaledonica | *F. nitidifolia* Bureau (1872) | New Caledonia | aus |
| Glandulosae | F. nervosa | *F. novae-georgiae* Corner (1967) | Solomon Islands (Bougainville, Choiseul, Guadalcanal, Kolombangara, Malaita, New Georgia, Ranongga, Rendova, San Cristobal, Santa Isabel, Tetepari, Ulawa) | aus |
| Glandulosae | F. austrocaledonica | *F. otophora* Corner & Guillaumin (1959) | New Caledonia | aus |
| Glandulosae | F. austrocaledonica | *F. otophoroides* Corner (1975) | New Caledonia | aus |
| Glandulosae | F. nervosa | *F. pachysycia* Corner (1960) | Papua New Guinea | aus |
| Glandulosae | F. austrocaledonica | *F. pancheriana* Bureau (1872) | New Caledonia | aus |
| Glandulosae | F. nervosa | *F. polyantha* Warb. (1900) | Philippines (Bohol, Luzon, Leyte, Mindanao); Indonesia (Moluccas, Irian Jaya); Papua New Guinea (PNG, New Britain, New Ireland, Bougainville); Solomon Islands (Bougainville, Big Nggela, Guadalcanal, Kolombangara, Malaita, Malaupaina, Santa Isabel) | ori aus |
| Glandulosae | F. nervosa | *F. pseudojaca* Corner (1960) | Indonesia (Irian Jaya); Papua New Guinea | aus |
| Glandulosae | F. austrocaledonica | *F. pteroporum* Guillaumin (1967) | New Caledonia | aus |
| Glandulosae | F. austrocaledonica | *F. racemigera* Bureau (1872) | New Caledonia | aus |
| Glandulosae | F. nervosa | *F. saruensis* C.C. Berg (2003) | Papua New Guinea | aus |
| Glandulosae | F. nervosa | *F. sclerosycia* C.C. Berg (2003) | Papua New Guinea | aus |
| Glandulosae | F. nervosa | *F. setulosa* C.C. Berg (2002) | Solomon Islands (Choiseul) | aus |
| Glandulosae | F. nervosa | *F. smithii* Horne ex (1883) | Fiji; Vanuatu (Aneityum) | aus |
| Glandulosae | F. nervosa | *F. subcaudata* C.C. Berg (2003) | Papua New Guinea | aus |
| Glandulosae | F. nervosa | *F. subnervosa* Corner (1960) | Indonesia (Irian Jaya); Papua New Guinea (PNG) | aus |
| Glandulosae | F. nervosa | *F. subtrinervia* Lauterb. & K. Schum. (1900) | Indonesia (Sulawesi, Irian Jaya); Papua New Guinea (PNG, New Britain); Solomon Islands (Guadalcanal, Malaita, Santa Isabel) | ori aus |
| Pedunculatae | F. albipila | *F. variifolia* Warb. (1904) | Sierra Leone; Guinea; Ivory Coast; Sudan; Nigeria; Cameroon; Central African Republic; Tanzania; Congo; Gabon; Democratic Republic of Congo; Angola; Uganda | afr |
| Pedunculatae | F. vasculosa | *F. vasculosa* Miquel (1848) | Myanmar; China; Vietnam; Thailand; Laos; Malaysia (Peninsula, Sarawak); Indonesia (Kalimantan, Java, Sumatra ) | ori |
| Glandulosae | F. austrocaledonica | *F. versicolor* Bureau (1872) | New Caledonia | aus |
| Glandulosae | F. austrocaledonica | *F. vieillardiana* Bureau (1872) | New Caledonia | aus |
| Glandulosae | F. austrocaledonica | *F. webbiana* Miq. (1867) | New Caledonia | aus |
| Glandulosae | F. austrocaledonica | *Ficus* sp., new species | New Caledonia | aus |
