# Supplementary material for: Testing the Emergence of New Caledonia: Fig Wasp Mutualism as a Case Study and a Review of Evidence
Source: PLoS One. 2012 Feb 22;7(2):e30941. doi: 10.1371/journal.pone.0030941 (PMC3285151; doi:10.1371/journal.pone.0030941)
Supplement: Table S2 — List of Dolichoris and outgroup species included in this study. (DOC) [file pone.0030941.s002.doc]

**Table S2 : List of *Dolichoris* and outgroup species included in this study.**

Stars indicate probable misidentifications of host *Ficus* species in Genbank database.

| Voucher number | Genus | species | Host ficus species | Country | Province | COI | Cytb | EF | Wg | 28S | 18S |
| --- | --- | --- | --- | --- | --- | --- | --- | --- | --- | --- | --- |
| 0868_01x | *Dolichoris* | *nervosae* | *Ficus nervosa* | China | Yunnan | JN103244 | JN103135 | JN102993 | JN102754 | JN102824 | JN102899 |
| 1228_01x | *Dolichoris* | sp. 14 | *Ficus racemigera* | New Caledonia | Grande Terre | JQ256521 | ∅ | ∅ | ∅ | JN623544 / JN623876 | JN623054 |
| 1229_01x | *Dolichoris* | sp. 08 | *Ficus habrophylla* | New Caledonia | Grande Terre | GQ367869 | GQ367966 | JN102996 | GQ368061 | GQ367677 / GQ367774 | GQ367575 |
| 1230_01x | *Dolichoris* | sp. 08 | *Ficus habrophylla* | New Caledonia | Grande Terre | JQ256522 | JQ256706 | JQ256657 | JQ256614 | JQ256566 / JQ256590 | JQ256751 |
| 1231_01x | *Dolichoris* | sp. 08 | *Ficus habrophylla* | New Caledonia | Grande Terre | JQ256523 | JQ256707 | JQ256658 | JQ256615 | JQ256567 / JQ256591 | JQ256752 |
| 1232_01x | *Dolichoris* | sp. 03 | *Ficus austrocaledonica* | New Caledonia | Grande Terre | GQ367870 | GQ367967 | JQ256659 | GQ368062 | GQ367678 / GQ367775 | GQ367576 |
| 1233_01x | *Dolichoris* | sp. 03 | *Ficus austrocaledonica* | New Caledonia | Grande Terre | JQ256524 | JQ256708 | JQ256660 | JQ256616 | JN623543 / JN623875 | JN623053 |
| 1234_01x | *Dolichoris* | sp. 01 | *Ficus asperula* | New Caledonia | Grande Terre | JQ256525 | JQ256709 | JQ256661 | JQ256617 | JN623545 / JN623877 | JN623055 |
| 1234_01y | *Dolichoris* | sp. 01 | *Ficus asperula* | New Caledonia | Grande Terre | JQ256526 | JQ256710 | JQ256662 | JQ256618 | JN623545 / JN623877 | JN623055 |
| 1235_01x | *Dolichoris* | sp. 08 | *Ficus habrophylla* | New Caledonia | Grande Terre | ∅ | JQ256711 | ∅ | JQ256619 | JQ256568 / JQ256592 | JQ256753 |
| 1236_01x | *Dolichoris* | sp. 01 | *Ficus asperula* | New Caledonia | Grande Terre | JQ256527 | JQ256712 | JQ256663 | JQ256620 | JN623546 / JN623878 | JQ256754 |
| 1237_01x | *Dolichoris* | sp. 01 | *Ficus asperula* | New Caledonia | Grande Terre | GQ367871 | GQ367968 | JQ256664 | GQ368063 | GQ367679 / GQ367776 | GQ367577 |
| 1238_01x | *Dolichoris* | sp. 10 | *Ficus nitidifolia* | New Caledonia | Grande Terre | JQ256528 | ∅ | JQ256665 | ∅ | GQ367680 / GQ367777 | GQ367578 |
| 1238_01y | *Dolichoris* | sp. 10 | *Ficus nitidifolia* | New Caledonia | Grande Terre | JQ256529 | ∅ | JQ256666 | ∅ | GQ367680 / GQ367777 | GQ367578 |
| 1239_01x | *Dolichoris* | sp. 01 | *Ficus* sp. | New Caledonia | Grande Terre | JQ256530 | JQ256713 | JQ256667 | JQ256621 | JN623536 / JN623868 | JN623056 |
| 1245_01x | *Dolichoris* | sp. 07 | *Ficus granatum minor* | Vanuatu Islands |  | JQ256531 | JQ256714 | JQ256668 | JQ256622 | JQ256569 / JQ256593 | JQ256755 |
| 1248_01x | *Dolichoris* | sp. 07 | *Ficus granatum minor* | Vanuatu Islands |  | JQ256532 | JQ256715 | JQ256669 | JQ256623 | JN623538 / JN623870 | JN623047 |
| 1250_01x | *Dolichoris* | sp. 06 | *Ficus granatum* | Vanuatu Islands |  | JQ256533 | JQ256716 | JQ256670 | JQ256624 | JQ256570 / JQ256594 | JQ256756 |
| 1250_01y | *Dolichoris* | sp. 06 | *Ficus granatum* | Vanuatu Islands |  | JQ256534 | ∅ | JQ256671 | JQ256625 | JQ256570 / JQ256594 | JQ256756 |
| 1252_02 | *Pleistodontes* | sp. nr blandus | *Ficus glandifera* | Vanuatu Islands |  | GQ367875 | ∅ | JN102998 | GQ368067 | GQ367682 / GQ367779 | GQ367581 |
| 1258_01x | *Dolichoris* | sp. 08 | *Ficus habrophylla variegata* | New Caledonia | Maré | ∅ | JQ256717 | JQ256672 | JQ256626 | JQ256571 / JQ256595 | JQ256757 |
| 1260_01x | *Dolichoris* | sp. 08 | *Ficus habrophylla variegata* | New Caledonia | Maré | JQ256535 | JQ256718 | JQ256673 | JQ256627 | JQ256572 / JQ256596 | JQ256758 |
| 1265_01x | *Dolichoris* | sp. 09 | *Ficus lifouensis* | New Caledonia | Ouvéa | JQ256536 | JQ256719 | JQ256674 | JQ256628 | JN623539 / JN623871 | JN623048 |
| 1269_01x | *Dolichoris* | sp. 14 | *Ficus racemigera* | New Caledonia | Grande Terre | ∅ | JQ256720 | JQ256675 | JQ256629 | JQ256573 / JQ256597 | JQ256759 |
| 1271_01x | *Dolichoris* | sp. 04 | *Ficus cataractarum* | New Caledonia | Grande Terre | JQ256537 | JQ256721 | JQ256676 | JQ256630 | JQ256574 / JQ256598 | JQ256760 |
| 1272_01x | *Dolichoris* | sp. 12 | *Ficus pancheriana* | New Caledonia | Grande Terre | JQ256538 | ∅ | JQ256677 | JQ256631 | JQ256575 / JQ256599 | ∅ |
| 1272_01y | *Dolichoris* | sp. 12 | *Ficus pancheriana* | New Caledonia | Grande Terre | JQ256539 | ∅ | JQ256678 | JQ256632 | JQ256575 / JQ256599 | JQ256761 |
| 1273_01x | *Dolichoris* | sp. 11 | *Ficus otophora* | New Caledonia | Grande Terre | JQ256540 | JQ256722 | JQ256679 | JQ256633 | JQ256576 / JQ256600 | JQ256762 |
| 1273_01y | *Dolichoris* | sp. 11 | *Ficus otophora* | New Caledonia | Grande Terre | JQ256541 | ∅ | JQ256680 | ∅ | JQ256576 / JQ256600 | JQ256762 |
| 1274_01x | *Dolichoris* | sp. 03 | *Ficus austrocaledonica* | New Caledonia | Grande Terre | JQ256542 | JQ256723 | JQ256681 | JQ256634 | JQ256577 / JQ256601 | JQ256763 |
| 1275_01x | *Dolichoris* | sp. 14 | *Ficus racemigera* | New Caledonia | Grande Terre | ∅ | JQ256724 | JQ256682 | JQ256635 | JN623542 / JN623874 | JN623052 |
| 1276_01x | *Dolichoris* | sp. 14 | *Ficus racemigera* | New Caledonia | Grande Terre | GQ367878 | GQ367973 | JN103000 | GQ368070 | GQ367685 / GQ367782 | GQ367584 |
| 1277_01x | *Dolichoris* | sp. 04 | *Ficus cataractarum* | New Caledonia | Grande Terre | ∅ | JQ256725 | JQ256683 | JQ256636 | JQ256578 / JQ256602 | JQ256764 |
| 1278_01x | *Dolichoris* | sp. 12 | *Ficus pancheriana* | New Caledonia | Grande Terre | ∅ | ∅ | JQ256684 | JQ256637 | JQ256579 / JQ256603 | JQ256765 |
| 1280_01x | *Dolichoris* | sp. 11 | *Ficus otophora* | New Caledonia | Grande Terre | JQ256543 | JQ256726 | JQ256685 | JQ256638 | JQ256580 / JQ256604 | JQ256766 |
| 1280_01y | *Dolichoris* | sp. 11 | *Ficus otophora* | New Caledonia | Grande Terre | JQ256544 | ∅ | JQ256686 | ∅ | JQ256580 / JQ256604 | JQ256766 |
| 1281_01x | *Dolichoris* | sp. 16 | *Ficus versicolor* | New Caledonia | Grande Terre | JQ256545 | JQ256727 | JQ256687 | JQ256639 | JQ256581 / JQ256605 | JQ256767 |
| 1283_01x | *Dolichoris* | sp. 02 | *Ficus asperula* | New Caledonia | Grande Terre | JQ256546 | JQ256728 | JQ256688 | JQ256640 | JN623540 / JN623872 | JN623049 |
| 1283_01y | *Dolichoris* | sp. 02 | *Ficus asperula* | New Caledonia | Grande Terre | JQ256547 | JQ256729 | JQ256689 | JQ256641 | JN623540 / JN623872 | JN623049 |
| 1284_01x | *Dolichoris* | sp. 15 | *Ficus* sp. | New Caledonia | Grande Terre | JQ256548 | JQ256730 | ∅ | JQ256642 | JQ256582 / JQ256606 | JQ256768 |
| 1286_01x | *Dolichoris* | sp. 16 | *Ficus versicolor* | New Caledonia | Grande Terre | JQ256549 | JQ256731 | JQ256690 | JQ256643 | JQ256583 / JQ256607 | JQ256769 |
| 1288_01x | *Dolichoris* | sp. 02 | *Ficus asperula* | New Caledonia | Grande Terre | ∅ | JQ256732 | JQ256691 | JQ256644 | GQ367686 / GQ367783 | GQ367585 |
| 1288_01y | *Dolichoris* | sp. 02 | *Ficus asperula* | New Caledonia | Grande Terre | GQ367879 | GQ367974 | JQ256692 | GQ368071 | GQ367686 / GQ367783 | GQ367585 |
| 1290_01x | *Dolichoris* | sp. 05 | *Ficus dzumacensis* | New Caledonia | Grande Terre | ∅ | JQ256733 | ∅ | ∅ | JQ256584 / JQ256608 | JQ256770 |
| 1331_01 | *Tetrapus* | *americanus* | *Ficus maxima* | French Guiana |  | GQ367880 | GQ367975 | JN103005 | GQ368072 | GQ367687 / GQ367784 | ∅ |
| 1334_01x | *Dolichoris* | sp. 13 | *Ficus pteroporum* | New Caledonia | Grande Terre | ∅ | JQ256734 | JQ256693 | JQ256645 | JQ256585 / JQ256609 | JQ256771 |
| 1334_01y | *Dolichoris* | sp. 13 | *Ficus pteroporum* | New Caledonia | Grande Terre | ∅ | JQ256735 | JQ256694 | JQ256646 | JQ256585 / JQ256609 | JQ256771 |
| 1479_01x | *Dolichoris* | sp. 06 | *Ficus granatum* | Vanuatu Islands |  | JQ256550 | JQ256736 | JQ256695 | JQ256647 | JQ256610 | JQ256772 |
| 1479_01y | *Dolichoris* | sp. 06 | *Ficus granatum* | Vanuatu Islands |  | JQ256551 | JQ256737 | JQ256696 | JQ256648 | JQ256610 | JQ256772 |
| 1536_01 | *Blastophaga* | *psenes* | *Ficus carica* | France | Herault | GQ367895 | ∅ | JN103013 | GQ368085 | GQ367703 / GQ367799 | GQ367602 |
| 1554_01 | *Pleistodontes* | *regalis* | *Ficus pleurocarpa* | Australia | Queensland | JN103255 | JN103153 | JN103014 | ∅ | JN102691 / JN102837 | JN102910 |
| 1634_01x | *Dolichoris* | *malabarensis* | *Ficus callosa* | China | Yunnan | JQ256552 | JQ256738 | ∅ | JQ256649 | JQ256611 | ∅ |
| 1634_01y | *Dolichoris* | *malabarensis* | *Ficus callosa* | China | Yunnan | JQ256553 | JQ256739 | ∅ | ∅ | JQ256611 | ∅ |
| 1682_01 | *Pegoscapus* | *bagataensis* | *Ficus andicola* | Colombia | Cundinamarca | GQ367905 | GQ368001 | JN103025 | GQ368096 | GQ367714 / GQ367810 | GQ367613 |
| 1810_01 | *Ceratosolen* | *flabellatus* | *Ficus sur* | Burkina Faso |  | JN103267 | JN103173 | JN103036 | JN102770 | JN102846 | JN102926 |
| 1816_01 | *Ceratosolen* | *stupefactus* | *Ficus tiliifolia* | Madagascar |  | GQ367914 | GQ368011 | JN103038 | GQ368107 | GQ367725 / GQ367819 | GQ367623 |
| 1953_01 | *Tetrapus* | sp. | *Ficus tonduzii* | Costa Rica | Puntarenas | GQ367942 | GQ368039 | JN103060 | GQ368135 | GQ367752 / GQ367846 | GQ367652 |
| 2050_11x | *Dolichoris* | cf *umbilicata* | *Ficus polyantha* | Papua New Guinea | Sepik East | JQ256554 | JQ256740 | ∅ | JQ256650 | JQ256586 / JQ256612 | ∅ |
| 2050_11y | *Dolichoris* | cf *umbilicata* | *Ficus polyantha* | Papua New Guinea | Sepik East | JQ256555 | ∅ | ∅ | ∅ | JQ256586 / JQ256612 | ∅ |
| 2075_01x | *Dolichoris* | sp. 17 | *Ficus matanoensis* | Indonesia | Sulawesi | JQ256556 | JQ256741 | JQ256697 | ∅ | JQ256587 / JQ256613 | JQ256773 |
| 2075_01y | *Dolichoris* | sp. 17 | *Ficus matanoensis* | Indonesia | Sulawesi | JQ256557 | JQ256742 | JQ256698 | ∅ | JQ256587 / JQ256613 | JQ256773 |
| 2092_01x | *Dolichoris* | cf *valentinae* | *Ficus nervosa pubinervis* | Indonesia | Sulawesi | ∅ | JQ256743 | ∅ | ∅ | ∅ | ∅ |
| 2092_02x | *Dolichoris* | cf *valentinae* | *Ficus nervosa pubinervis* | Indonesia | Sulawesi | JQ256558 | JQ256744 | JQ256699 | JQ256651 | ∅ | ∅ |
| 2132_01x | *Dolichoris* | sp. 03 | *Ficus habrophylla** | New Caledonia | Grande Terre | JQ256559 | JQ256745 | JQ256700 | JQ256652 | JN623537 / JN623869 | JN623050 |
| 2132_01y | *Dolichoris* | sp. 03 | *Ficus habrophylla** | New Caledonia | Grande Terre | JQ256560 | JQ256746 | JQ256701 | JQ256653 | JN623537 / JN623869 | JN623050 |
| 2133_01x | *Dolichoris* | sp. 03 | *Ficus habrophylla** | New Caledonia | Grande Terre | JQ256561 | JQ256747 | JQ256702 | JQ256654 | JN623541 / JN623873 | JN623051 |
| 2133_01y | *Dolichoris* | sp. 03 | *Ficus habrophylla** | New Caledonia | Grande Terre | JQ256562 | JQ256748 | JQ256703 | JQ256655 | JN623541 / JN623873 | JN623051 |
| 2136_01 | *Pegoscapus* | sp. | *Ficus eximia* | Brazil | Amazonas | JN103281 | JN103187 | JN103076 | JN102780 | JN102856 | JN102940 |
| 2335_01x | *Dolichoris* | sp. 19 | *Ficus* sp. | Indonesia | Papua Barat | JQ256563 | JQ256749 | JQ256704 | JQ256656 | JN102875 / JN102730 | JN102959 |
| 2335_01y | *Dolichoris* | sp. 19 | *Ficus* sp. | Indonesia | Papua Barat | JN103301 | JN103210 | JN103097 | JN102792 | JN102875 / JN102730 | JN102959 |
| 2352_01x | *Dolichoris* | sp. 18 | *Ficus polyantha* | Indonesia | Papua Barat | JQ256564 | JQ256750 | JQ256705 | JN102794 | JN102877 / JN102732 | JN102961 |
| 2352_01y | *Dolichoris* | sp. 18 | *Ficus polyantha* | Indonesia | Papua Barat | JN103303 | JN103212 | JN103099 | ∅ | JN102877 / JN102732 | JN102961 |
| 2368_01x | *Dolichoris* | sp. 20 | *Ficus subtrinervia* | Indonesia | Papua Barat | JN103307 | ∅ | JN103102 | JN102795 | JQ256588 | JN102964 |
| 2670_02x | *Dolichoris* | *flabellatus* | *Ficus dicranostyla* | Mali |  | JQ256565 | ∅ | ∅ | ∅ | JQ256589 | ∅ |
| 8059_01a | *Dolichoris* | *inornata* | *Ficus edelfeltii* | Papua New Guinea |  | AF200395 | ∅ | ∅ | ∅ | AF200395 | ∅ |
| 8060_01a | *Dolichoris* | *vasculosae* | *Ficus vasculosa* | Malaysia |  |  | ∅ | ∅ | ∅ | AY616555 | ∅ |
| 8228_01a | *Dolichoris* | sp. 21 | *Ficus subtrinervia* | Papua New Guinea |  | AF200394 | ∅ | ∅ | ∅ | AY616553 | ∅ |
| 8229_01a | *Dolichoris* | sp. 06 | *Ficus granatum* | Vanuatu Islands |  | AY642461 | ∅ | ∅ | ∅ | AY616556 | ∅ |
