# Supplementary material for: Testing the Emergence of New Caledonia: Fig Wasp Mutualism as a Case Study and a Review of Evidence
Source: PLoS One. 2012 Feb 22;7(2):e30941. doi: 10.1371/journal.pone.0030941 (PMC3285151; doi:10.1371/journal.pone.0030941)
Supplement: Table S3 — List of Oreosycea and outgroup species included in this study. (DOC) [file pone.0030941.s003.doc]

**Table S3**: **List of *Oreosycea* and outgroup species included in this study**.

| **Subgenus** | **Section** | **Species** | **ITS** | **ETS** | **G3pdh** |
| --- | --- | --- | --- | --- | --- |
| *Pharmacosycea* | *Oreosycea* | *F. callosa* | AY063565 | AY063526 | EF092367 |
| *Pharmacosycea* | *Oreosycea* | *F. dicranostyla* | EU091566 | EU084407 | EF092368 |
| *Pharmacosycea* | *Oreosycea* | *F. edelfeltii* | AF165385 | AY730209 |  |
| *Pharmacosycea* | *Oreosycea* | *F. habrophylla* | EU091567 |  |  |
| *Pharmacosycea* | *Oreosycea* | *F. hombroniana* |  |  | EF092369 |
| *Pharmacosycea* | *Oreosycea* | *F. nervosa* | EU091570 | EU084410 | EU087615 |
| *Pharmacosycea* | *Oreosycea* | *F. polyantha* | EU091571 |  | EU087616 |
| *Pharmacosycea* | *Oreosycea* | *F. racemigera* | AY063587 | AY063554 |  |
| *Pharmacosycea* | *Oreosycea* | *F. subtrinervia* | AY730119 | EU084411 | EU087617 |
| *Pharmacosycea* | *Oreosycea* | *F. vasculosa* | EU091572 | EU084412 |  |
| *Pharmacosycea* | *Oreosycea* | *F. albipila* |  |  | EF092366 |
| *Pharmacosycea* | *Pharmacosycea* | *F. insipida* | AF165390 | AY063549 | EU08986 |
| *Pharmacosycea* | *Pharmacosycea* | *F. maxima* | AY063595 | AY063551 | EU089876 |
| *Urostigma* | *Malvanthera* | *F. glandifera* | AY730113 | AY730202 | EF092361 |
| *Urostigma* | *Malvanthera* | *F. macrophylla* | AY063571 | AY063532 | EF538792 |
| *Urostigma* | *Americana* | *F. americana* | AY730070 | AY730158 | EF092339 |
| *Urostigma* | *Americana* | *F. eximia* | AY730079 | AY730167 | EF092344 |
| *Sycomorus* | *Sycomorus* | *F. mucuso* | AY730120 | AY730210 | EF092372 |
| *Sycomorus* | *Sycomorus* | *F. sycomorus* | AY063575 | AY063536 |  |
| *Ficus* | *Ficus* | *F. carica* | EU091637 | EU084464 |  |
