# Supplementary material for: Testing the Emergence of New Caledonia: Fig Wasp Mutualism as a Case Study and a Review of Evidence
Source: PLoS One. 2012 Feb 22;7(2):e30941. doi: 10.1371/journal.pone.0030941 (PMC3285151; doi:10.1371/journal.pone.0030941)
Supplement: Table S4 — Details of studies included in the review of New Caledonia's biogeography. (DOC) [file pone.0030941.s004.doc]

**Table S4. Details of studies included in the review of New Caledonia biogeography.**

Ant= Antarctica ; Af = Africa, Aus = Australia, Mada = Madagascar, Mal = Malesia, NC = New Caledonia, NG = New Guinea, NZ = New Zealand, Pac= Pacific islands, Pat= Patagonia, SAm = South America, Sol = Solomon Islands, Van = Vanuatu.

HPD : 95% highest posterior density interval.

|  | **Level of endemicity** | **Taxa name** | **Abbreviation used in Figure 1 (Main Text)** | **Stem group estimates**  mean age Ma (95% HPD) | **Crown group estimates**  mean age Ma (95% HPD) | **Details / sister taxa relationships** | **Proposed origin of NC taxa or closest outgroup node** | **study** |
| --- | --- | --- | --- | --- | --- | --- | --- | --- |
| **Plants**  24 taxa | Genus (1 sp.) | *Nemuaron vieillardii*  (Atherospermataceae) | *Nemuaron* | 37.0 (55.0 - 19.0) |  | split from *Atherosperma moschatum*, Aus+Tasmania | unknown, dipersal from Aus or Ant is proposed | [1] |
|  | Species | *Acridocarpus austrocaledonicus*  (Malpighiaceae) | *Acridocarpus* | 8.0 (9.0 - 7.0) |  | split from other *Acridocarpus* spp., Mada | Mada (the species is nested within a Malagasy clade) | [2] |
|  | Species | *Brassospora* spp.  (Nothofagaceae) | *Brassospora* | 27.5 (52.0 - 3.0) |  | crown extant *Brassospora* spp., NG+NC | unknown, dispersal from Aus or NZ is proposed | [3] |
|  | Genera and Species | *Beccariella* spp.  *Planchonella* spp.  *Leptostylis* clade  *Pichonia* spp.  (Sapotaceae) | *Beccariella*  *Planchonella*  *Leptostylis*  *Pichonia* | ∼48.3  43.1 (49.5-36.8)  38.1 (44.3-31.9)  31.2 (38.2-24.2) | 12.3 (16.6-8.0)  39.3 (45.2-32.3)  23.8 (29.7-18.7)  16.7 (22.1-11.3) | Aus | Aus | [4] |
|  | Genus (1 sp.) | *Sparattosyce dioica*  (Moraceae) | *Sparattosyce* | ∼45.0 |  | split from *Antiaropsis decipiens*, NG | unknown | [5] |
|  | Genus (13 spp.) | *Beauprea montana*  (Proteaceae) | *Beauprea* | ∼80.0 |  | split from *Protea cynaroides,* Afr | Aus or Af (Closest outgroup node) | [6] |
|  | Species | *Santalum austrocaledonicum*,  (Santalaceae) | *Santalum* | 2.2 (2.5 - 1.9) | 1.3 (1.5 - 1.0) | split from *Santalum lanceolatum*, Aus | Aus (inferred by ancestral area reconstruction) | [7] |
|  | Genus (6 spp.) | *Virotia* (2 spp.)  (Proteaceae) | *Virotia* | 18.0 (27.2 - 8.7) |  | crown Virotiinae, China+Mal+Aus | Aus (inferred by ancestral area reconstruction) | [8] |
|  | Genus (1 sp.) | *Sleumerodendron austrocaledonicum*  (Proteaceae) | *Sleumerodendron* | 14.9 (23.5 - 6.3) |  | split from *Euplassa* spp., SAm | Ambiguous Aus/SAm (inferred by ancestral area reconstruction) | [8] |
|  | Genus (4 ssp.) | *Kermadecia* (3 spp)  (Proteaceae) | *Kermadecia* | 10.3 (16.9 - 3.7) |  | split from *Turrillia* spp., Fiji + Van | Ambiguous Aus/SAm (inferred by ancestral area reconstruction) | [8] |
|  | Species + Genus (5spp.) | *Micromelum* and *Murraya spp*+ genus *Oxanthera.*  (Rutaceae) | Aurantioideae | 19.8 (28.2 - 12.1) |  | crown Aurantioideae | unknow, several dispersal events from Aus and SE Asia are proposed | [9] |
|  | Species | *Piper austrocaledonicum*  (Piperaceae) | *Piper* | ∼28.7 |  | split from other *Piper* spp., Asia | Asia (inferred by ancestral area reconstruction but Aus region includes NC) | [10] |
|  | Species | Phyllantheae spp  (Phyllanthaceae) | Phyllantheae | ∼34.0 | ∼20.0 | split from other Phyllantheae spp. | unknown | [11] |
|  | Species | *Zygogynum* spp.  (Winteraceae) | *Zygogynum* | 57.6 (69.0 - 46.2) | 29.7 (35.2 - 24.2) | split from *Pseudowintera colorata*, NZ | Ambiguous : Aus/NG/NZ | [12] |
|  | Species | *Soulamea moratti*  (Simaroubaceae*)* | *Soulamea* | 16.5 (22.6 - 10.4) |  | split from other *Soulamea* spp., Mada, Asia, Pac | Asia/SE Asia (inferred by ancestral area reconstruction but Aus region includes NC) | [13] |
|  | Family (1 sp.) | *Amborella trichopoda*  Amborellaceae | Amborellaceae | 190.0 (236.5-143.5) |  | split from Nymphaeales | unknown | [14] |
|  | Species | *Hedycarya cupulata*  (Monimiaceae) | *Hedycarya* | 11.1 (17.8 - 4.4) |  | split from other *Hedycarya* spp. Aus | Aus(inferred by ancestral area reconstruction) | [15] |
|  | Genus (1 sp.) | *Kibairopsis caledonica*  (Monimiaceae) | *Kibairopsis* | 17.2 (27.1 - 7.3) |  | split from *Hedycarya arborea,* NZ | Aus(inferred by ancestral area reconstruction) | [15] |
|  | Family (2 spp) | *Oncotheca balansae*  (Oncothecaceae) | Oncothecaceae | 98.0 (109.0 - 87.0) |  | split from Garryales-Lamiales clade | unknown | [14] |
|  | Genus (1 sp.) | *Arillastrum gummiferum*  (Myrtaceae) | *Arillastrum* | ∼44.4 |  | split from *Angophora hispida*, Aus | Aus (closest outgroup node) | [16] |
|  | Species | *Dacrydium* spp.  (Podocarpaceae) | *Dacrydium* | 10.0 (20.0-5.0) | ∼5.63 | Split from *Dacrydium* spp. (Fiji) | unknown | [17] |
| **Vertebrates**  6 taxa | Genus (1 sp.) | *Nesogalaxias neocaledonicus*  (Galaxiidae) | *Nesogalaxias* | 7.8 (12.6 - 2.9) |  | split from *Galaxias brevipinnis*, NZ | NZ | [18] |
|  | Family (1 sp.) | *Rhynochetos jubatus*  (Rhynochetidae) | Rhynochetidae | ∼31.0 |  | split from *Eurypyga helias*, SAm | unknown | [19] |
|  | Genera and Species | *Eugongylus* group  (Scincidae) | *Eugongylus* | 26.7 (40.7 - 12.7) |  | split from other *Eugongylu*s group species, Aus ? | unknown, dipersal from Aus is proposed | [20] |
|  | Genus (2 spp.) | *Eunymphicus uvaeensis*  (Psittacidae) | *Eunymphicus* | 32.2 (45.9 - 18.5) |  | crown Platycercini tribe (age overestimation), sister to *Cyanoramphus auriceps*, NZ | unknown, Fiji (closest outgroup node) | [21] |
|  | Genera (12 spp and 7 spp resp.) | *Bavayia* + *Rhacodactylus* spp.  (Diplodactylidae) | *Bavayia* | 48.9 (69.8 – 27.9) | ∼9.6 | split from *Pseudothecadactlylus* spp., Aus | Aus | [22] |
|  | Species | *Coracina* *analis*.  (Campephagidae) | *Coracina* | 10.3 (12.6 - 7.9) |  | split from other *Coracina* spp., Philippine archipelago | Ambiguous Aus/Philippines/Wallacea (inferred by ancestral area reconstruction) | [23] |
| **Arthropods** 24 taxa | Species | *Pauropsalta johanae* + *Myersalna depicta*  (Cicadidae) | *Pauropsalta* | ∼10.2 | ∼8.9 | split from other Cicadas spp., Aus | Aus/NZ (closest outgroup node) | [24] |
|  | Species | *Angustonicus* spp.  (Blattidae) | *Angustonicus* | 2.7 (4.0 - 1.3) |  | split from other *Angustonicus* spp., Loyalty Islands | Aus | [25] |
|  | Species | *Paratya* spp.  (Atyidae) | *Paratya* | 5.5 (8.6 - 2.3) |  | split from other *Paratya* spp., Norforlk and Lord Howe islands | Aus (closest outgroup node) | [26] |
|  | Family (1 Genus, 13 spp.) | Troglosironidae (4 spp.)  (Opiliones) | Troglosironidae | 172.5 (221.0 – 124.0) | 38.5 (49.0 - 28.0) | split from Neogoveidae spp. (SAm, W Af) | unknown | [27]* |
|  | Species | *Papuadytes spp.*  (Dytiscidae) | *Papuadytes 2*  *Papuadytes 1* | 2 colonisations :  10.3 (calibration)  14.5 (15.5 - 13.5) | 9.3 (10.9 -7.8)  ∼10.9 | split from other *Papuadytes* spp., Aus | Aus | [28] |
|  | Species | *Rhantus*  (Dytiscidae) | *Rhantus* | ∼7.9 | ∼2.3 | split from *Carabdytes upin*, NG | unknown | [29] |
|  | Species | *Dolichoris*  *(*Agaonidae) | *Dolichoris 1* | ∼64.0 | ∼21.0 (16.0 - 57.0) | split from other *Dolichoris* spp., China+Mal+NG | unknown | [30] |
|  | Species | *Lanceocercata* clade  (Phasmatodea) | *Lanceocercata 2* | ∼22.0 |  | split from *Megacrania* spp., Aus | unknown | [31] |
|  | Species + Genus | *Orthopsyche* + *Caledopsyche*  (Hydropsychidae) | *Orthopsyche* | 29.7 | 28.2 (22.4 - 32.5 ) | split from other Hydropsychidae spp., NZ, NG, Fiji ? (35.0 - 22.4 Ma)  and ingroup Hydropsychidae (45.8 - 43.6 Ma) | unknown (NZ ?) | [32] |
|  | Genera | *Lanceocercata* clade  (Phasmatodea) | *Lanceocercata 1* | 44.1 (31.1 - 59.4) | 41.06 (29.05 - 55.40). | split from the other *Lanceocercata* clades, Aus+Pac | unknown (Aus+Pac ?) | [33] |
|  | Species | *Arsipoda* spp.  (Chrysomelidae) | *Arsipoda* |  | 24.1 - 6.4 | crown group NC | unknown | [34] |
|  | Species | *Pheidole* spp.  (Formicidae) | *Pheidole* | 14.9 (17.0 - 13.1) | ∼9 8 | Split from other *Pheidole* spp., NG, Aus | Aus+Fiji+NG (closest outgroup node) | [35] |
|  | Species | *Lordomyrma* spp.  (Formicidae*)* | *Lordomyrma* | 13.5 | 9.0 | Split from another *Lordomyrma* sp. (undescribed)*,* Aus | Philippines+Mal (closest outgroup node) | [36] |
|  | Genus (1 sp.) | *Paratisiphone lyrnessa*  (Nymphalidae) | *Paratisiphone* | 28.6 (34.7 - 22.5) |  | split from *Tisiphone abeona*, Aus | unknown | [37] |
|  | Species | *Leptomyrmex*  (Formicidae) | *Leptomyrmex* | 10.5 (15.9-5.4) | 4.1 (6.9-1.4) | Split from other *Leptomyrmex* spp. (Aus) | Aus | [38] |
|  | Species | *Cryptops sarasini*  *Cryptops pictus*  (Cryptopidae) | *Cryptops 2*  *Cryptops 1* | 7.3 (19.1-0.6)  15.8 (34.0-5.0) | 11.7 (23.7-2.9) | Split from *C. spinipes* (Aus, NZ, Fiji, Sol, Cont Asia)  Split from *C. spinipes* and *C. sarasini* | unknown | [39] |
|  | Genus (20 spp.) | *Agnotecous* (5 spp.)  (Grylloidea) | *Agnotecous* | 15.3(19.3-9.4) | 10.6(16.3-5.3) | Split from *Lebinthus* spp. (Van) | Indo-Malaysia ? | [40] |
|  | Species | *Cardiodactylus novaeguinae*  (Grylloidea) | *Cardiodactylus* | 12.6(19.3-6.6) | 3.3(6.3-1.1) | *Cardiodactylus novaeguinae* species group (NG, Van, Loyalty Islands) | Indo-Malaysia ? | [40] |
|  | Genus (>80 spp.) | *Agmina* spp. (75 spp.)  (Ecnomidae) | *Agmina* | 36.6 (48.3-29.7) | 21.9 (24.6-16.8) | *Ecnomina legula* (Aus) *+ Caledomina* spp. (NC) | Aus | [41] |
|  | Genus (4 spp.) | *Caledomina* spp. (4 spp.)  (Ecnomidae) | *Caledomina* | 25.9 (38.2-21.4) | 9.5(13.2-6.4) | *Ecnomina legula* (Aus) | Aus | [41] |
|  | species | *Apsilochorema caledonicum* (Hydrobiosidae) | *Apsilochorema* | 15.3 (26.0–7.0) | 1.4 (2.82-0.38) | *Apsilochorema gisbum* (Aus) | Aus or Oriental region (inferred by ancestral area reconstruction) | [42] |
|  | species | *Dracophyllum* spp.  (Ericaceae) | *Dracophyllum* | 6.7 (9.7-4.0) | 5.2 (7.2-2.6) | unresolved | Aus ? | [43] |
|  | Genus (10 spp.) | *Gracilipsodes* spp.  (Leptoceridae) | *Gracilipsodes* |  | 14.4 | *Triplexa villa* (Aus) | unknown | [44 ,45] |
|  | species | *Helicopha* spp.  (Helicophidae) | *Helicopha* |  | 8.2 | *Helicopha* sp. (Aus) | unknown | [45,46 ] |
|  | Genus (> 9 spp.) | *Xanthochorema* spp. (> 9 spp.) (Hydrobiosidae) | *Xanthochorema* |  | 11.9 | *Psilochorema leptoharpax* (NZ) | unknown | [45,47] |

*The study by Sharma and Giribet [48] was not included in the present review because calibration used was based on Boyer *et al.*[27]

**REFERENCES**

1. Renner SS, Foreman DB, Murray D (2000) Timing transantarctic disjunctions in the Atherospermataceae (Laurales): Evidence from coding and noncoding chloroplast sequences. Syst Biol 49: 579-591.

2. Davis CC, Bell CD, Fritsch PW, Mathews S (2002) Phylogeny of *Acridocarpus*-*Brachylophon* (Malpighiaceae): implications for Tertiary tropical floras and afroasian biogeography. Evolution 56: 2395-2405.

3. Cook LG, Crisp MD (2005) Not so ancient: the extant crown group of *Nothofagus* represents a post-Gondwanan radiation. Proc R Soc Biol Sci Ser B 272: 2535-2544.

4. Bartish IV, Antonelli A, Richardson JE, Swenson U (2011) Vicariance or long-distance dispersal: historical biogeography of the pantropical subfamily Chrysophylloideae (Sapotaceae). J Biogeogr 38: 177-190.

5. Zerega NJC, Clement WL, Datwyler SL, Weiblen GD (2005) Biogeography and divergence times in the mulberry family (Moraceae). Mol Phylogenet Evol 37: 402-416.

6. Barker NP, Weston PH, Rutschmann F, Sauquet H (2007) Molecular dating of the 'Gondwanan' plant family Proteaceae is only partially congruent with the timing of the break-up of Gondwana. J Biogeogr 34: 2012-2027.

7. Harbaugh DT, Baldwin BG (2007) Phylogeny and biogeography of the Sandalwoods (Santalum, Santalaceae): repeated dispersals throughout the Pacific. Am J Bot 94: 1028–1040.

8. Mast AR, Willis CL, Jones EH, Downs KM, Weston PH (2008) A smaller Macadamia from a more vagile tribe: Inference of phylogenetic relationships, divergence times, and diaspore evolution in Macadamia and relatives (tribe Macadamieae; Proteaceae). Am J Bot 95: 843-870.

9. Pfeil BE, Crisp MD (2008) The Age and Biogeography of Citrus and the Orange Subfamily (Rutaceae: Aurantioideae) in Australasia and New Caledonia. Am J Bot 95: 1621-1631.

10. Smith JF, Stevens AC, Tepe EJ, Davidson C (2008) Placing the origin of two species-rich genera in the Late Cretaceous with later species divergence in the Tertiary: a phylogenetic, biogeographic and molecular dating analysis of *Piper* and *Peperomia* (Piperaceae). Plant Syst Evol 275: 9-30.

11. Kawakita A, Kato M (2009) Repeated independent evolution of obligate pollination mutualism in the Phyllantheae-Epicephala association. Proc R Soc Biol Sci Ser B 276: 417-426.

12. Marquínez X, Lohmann LG, Faria Salatino ML, Salatino A, González F (2009) Generic relationships and dating of lineages in Winteraceae based on nuclear (ITS) and plastid (*rp*S16 and *psb*A-*trn*H) sequence data. Mol Phylogenet Evol 53: 435-449.

13. Clayton JW, Soltis PS, Soltis DE (2009) Recent long-distance dispersal overshadows ancient biogeographical patterns in a pantropical angiosperm family (Simaroubaceae, Sapindales). Syst Biol 58: 395-410.

14. Smith SA, Beaulieu JM, Donoghue MJ (2010) An uncorrelated relaxed-clock analysis suggests an earlier origin for flowering plants. Proc Natl Acad Sci U S A 107: 5897–5902.

15. Renner SS, Strijk JS, Strasberg D, Thebaud C (2010) Biogeography of the Monimiaceae (Laurales): a role for East Gondwana and long-distance dispersal, but not West Gondwana. J Biogeogr 37: 1227-1238.

16. Sytsma KJ, Litt A, Zjhra ML, Pires C, Nepokroeff M, et al. (2004) Clades, clocks, and continents: historical and biogeographical analysis of Myrtaceae, Vochysiaceae, and relatives in the southern hemisphere. Int J Plant Sci 165: S85-S105.

17. Keppel G, Prentis P, Biffin E, Hodgskiss P, Tuisese S, et al. (2011) Diversification history and hybridisation of *Dacrydium* (Podocarpaceae) in remote Oceania. Aust J Bot 59: 262-273.

18. Waters JM, López A, Wallis GP (2000) Molecular Phylogenetics and Biogeography of Galaxiid Fishes (Osteichthyes: Galaxiidae): Dispersal, Vicariance, and the Position of *Lepidogalaxias salamandroides*. Syst Biol 49: 777-795.

19. Ericson PGP, Anderson CL, Britton T, Elzanowski A, Johansson US, et al. (2006) Diversification of Neoaves: integration of molecular sequence data and fossils. Biol Lett 2: 543-547.

20. Smith SA, Sadlier RA, Bauer AM, Austin CC, Jackman T (2007) Molecular phylogeny of the scincid lizards of New Caledonia and adjacent areas: evidence for a single origin of the endemic skinks of Tasmantis. Mol Phylogenet Evol 43: 1151–1166.

21. Wright TF, Schirtzinger EE, Matsumoto T, Eberhard JR, Graves GR, et al. (2008) A multilocus molecular phylogeny of the parrots (Psittaciformes): Support for a Gondwanan origin during the Cretaceous. Mol Biol Evol 25: 2141-2156.

22. Oliver PM, Sanders KL (2009) Molecular evidence for Gondwanan origins of multiple lineages within a diverse Australasian gecko radiation. J Biogeogr 36: 2044-2055.

23. Jønsson KA, Bowie RCK, Nylander JAA, Christidis L, Norman JA, et al. (2010) Biogeographical history of cuckoo-shrikes (Aves: Passeriformes): transoceanic colonization of Africa from Australo-Papua. J Biogeogr 37: 1767-1781.

24. Arensburger P, Buckley TR, Simon C, Moulds M, Holsinger KE (2004) Biogeography and phylogeny of the New Zealand cicada genera (Hemiptera : Cicadidae) based on nuclear and mitochondrial DNA data. J Biogeogr 31: 557-569.

25. Murienne J, Grandcolas P, Piulachs MD, Belles X, D'Haese C, et al. (2005) Evolution on a shaky piece of Gondwana: is local endemism recent in New Caledonia? Cladistics 21: 2-7.

26. Page TJ, Baker AM, Cook BD, Hughes JM (2005) Historical transoceanic dispersal of a freshwater shrimp: the colonization of the South Pacific by the genus Paratya (Atyidae). J Biogeogr 32: 581–593.

27. Boyer SL, Clouse RM, Benavides LR, Sharma P, Schwendinger PJ, et al. (2007) Biogeography of the world: a case study from cyphophthalmid Opiliones, a globally distributed group of arachnids. J Biogeogr 34: 2070-2085.

28. Balke M, Pons J, Ribera I, Sagata K, Vogler AP (2007) Infrequent and unidirectional colonization of hyperdiverse Papuadytes diving beetles in New Caledonia and New Guinea. Mol Phylogenet Evol 42: 505-516.

29. Balke M, Wewalka G, Alarie Y, Ribera I (2007) Molecular phylogeny of pacific island Colymbetinae: radiation of New Caledonian and Fijian species (Coleoptera, Dytiscidae). Zool Scr 36: 173-200.

30. Lopez-Vaamonde C, Cook JM, Rasplus J-Y, Machado CA, Weiblen G (2009) Molecular dating and biogeography of fig-pollinating wasps. Mol Phylogenet Evol 52: 715–726.

31. Buckley TR, Attanayake D, Bradler S (2009) Extreme convergence in stick insect evolution: phylogenetic placement of the Lord Howe Island tree lobster. Proceedings of the Royal Society (B) 276: 1055-1062.

32. Espeland M, Johanson KA (2010) The effect of environmental diversification on species diversification in New Caledonian caddisflies (Insecta: Trichoptera: Hydropsychidae). J Biogeogr 37: 879–890.

33. Buckley TR, Attanayake D, Nylander JAA, Bradler S (2010) The phylogenetic placement and biogeographical origins of the New Zealand stick insects (Phasmatodea). Syst Entomol 35: 207-225.

34. Gomez-Zurita J, Cardoso A, Jurado-Rivera JA, Jolivet P, Cazères S, et al. (2010) Discovery of new species of New Caledonian *Arsipoda* Erichson, 1842 (Coleoptera: Chrysomelidae) and insights on their ecology and evolution using DNA markers. J Nat Hist 44: 2557-2579.

35. Sarnat EM, Moreau CS (2011) Biogeography and morphological evolution in a Pacific island ant radiation. Mol Ecol 20: 114-130.

36. Lucky A, Sarnat EM (2010) Biogeography and diversification of the Pacific ant genus Lordomyrma Emery. J Biogeogr 37: 624-634.

37. Peña C, Nylin S, Wahlberg N (2011) The radiation of Satyrini butterflies (Nymphalidae: Satyrinae): a challenge for phylogenetic methods. Zool J Linn Soc 161: 64-87.

38. Lucky A (2011) Molecular phylogeny and biogeography of the spider ants, genus *Leptomyrmex* Mayr (Hymenoptera: Formicidae). Mol Phylogenet Evol 59: 281-292.

39. Murienne J, Edgecombe GD, Giribet G (2011) Comparative phylogeography of the centipedes *Cryptops pictus* and *C. niuensis* (Chilopoda) in New Caledonia, Fiji and Vanuatu. Org Divers Evol 11: 61-74.

40. Nattier R, Robillard T, Desutter-Grandcolas L, Couloux A, Grandcolas P (2011) Older than New Caledonia emergence? A molecular phylogenetic study of the eneopterine crickets (Orthoptera: Grylloidea). J Biogeogr in press.

41. Espeland M, Johanson KA (2010) The diversity and radiation of the largest monophyletic animal group on New Caledonia (Trichoptera: Ecnomidae: *Agmina*). J Evol Biol 23: 2112-2122.

42. Strandberg J, Johanson KA (2010) The historical biogeography of *Apsilochorema* (Trichoptera, Hydrobiosidae) revised, following molecular studies. J Zool Syst Evol Res 49: 110-118.

43. Wagstaff SJ, Dawson MI, Venter S, Munzinger J, Crayn DM, et al. (2010) Origin, diversification, and classification of the australasian genus *Dracophyllum* (Richeeae, Ericaceae). Ann Mo Bot Gard 97: 235-258.

44. Malm T, Johanson KA (2008) Revision of the New Caledonian endemic genus *Gracilipsodes* (Trichoptera: Leptoceridae: Grumichellini). Zool J Linn Soc 153: 425-452.

45. Espeland M, Murienne J (2011) Diversity dynamics in New Caledonia: towards the end of the museum model? BMC Evol Biol 11: 254.

46. Johanson KA, Keijsner M (2008) Phylogeny of the Helicophidae (Trichoptera), with emphasis on the New Caledonian species of *Helicopha*. Syst Entomol 33: 451-483.

47. Espeland M, Johanson KA, Hovmöller R (2008) Early *Xanthochorema* (Trichoptera, Insecta) radiations in New Caledonia originated on ultrabasic rocks. Mol Phylogenet Evol 48: 904-917.

48. Sharma P, Giribet G (2009) A relict in New Caledonia: phylogenetic relationships of the family Troglosironidae (Opiliones: Cyphophthalmi). Cladistics 25: 1-16.
